# Medical Opinion and Movement

**Published:** 1907-08-10

**Authors:** 


### Medical Opinion and Movement.

It is probable that the similarity between the *Spirochœta pallida* of syphilis and the trypanosome of sleeping sickness first suggested that atoxyl, which offers such promise of proving a specific for the latter malady, might also be used as a weapon against the former. The drug has been taken up especially by French syphilologists ,and already finds several advocates for its use in conjunction with mercury. Experience so far has not shown that it has the same specific action upon the disease as mercury, but it will probably be found to be a useful alternative for those cases in which mercury is tolerated badly. A note of warning has been raised, however, in regard to the use of atoxyl both in the treatment of sleeping sickness and syphilis. It is usually administered by hypodermic or intramuscular injections, and if given in too large doses is liable to give rise to acute symptoms of arsenical poisoning, which may suddenly assume alarming proportions. Neuritis and blindness may also follow the use of the drug. The plan usually followed is to give small doses at first, gradually increasing up to 50 or 60 minims of a 10 per cent. solution. In the treatment of sleeping sickness mercury is now being tried together with atoxyl, and Sir Patrick Monson has lately expressed approval of this combined treatment.

A great outcry is being raised that trachoma is greatly on the increase in this country, and that London in particular is "threatened with a plague of one of the most terrible maladies known to medical science," that it is "spreading to an alarming extent, and little or nothing is being done to check it." Trachoma is a disease which is especially prevalent among the Jews, and there is no doubt that the large immigration of pauper Russian Jews, which is continually taking place to our hospitable shores, tends to increase the number of infected individuals in our midst and adds greatly to the risks of an epidemic of the disease. We are not aware, however, that the disease has hitherto shown signs of assuming any such alarming proportions as some of our contemporaries would lead us to believe. Definite measures have been taken to prevent the spread of infection among children by providing special schools for those already afflicted with the disease, but there seems no doubt that the regulations to exclude immigrants suffering from the disease are not enforced with anything like the same stringency as holds in America. In consequence a large number of those who are refused admission into the latter country find an asylum here in London, Liverpool, and other cities. The disease is in no sense endemic, and could easily be stamped out from our midst by a proper exclusion of the undesirable alien afflicted with it.

At a recent meeting of the Académie de Médicine Professor Dieulafoy made some important observations upon the culture of the voice. He pointed out that the voice should be developed on physiological principles, but that neither in the schools nor even at the conservatoire did the masters possess the necessary technical knowledge for the purpose. He proposed that the Académie de Médicine should adopt the resolutions which had already been accepted by the Société de Laryngologie, namely: that no one should be allowed to teach elocution or singing unless he possessed the necessary knowledge of physiology and further, that the conservatoires should appoint one or two laryngologists to examine the pupils periodically at the commencement, during and on the completion of their studies. It would not be amiss if some such measures were also adopted in this country. The majority of professors of singing also profess a profound knowledge of the anatomy and physiology of the vocal organs, and impress their pupils and clients with an extensive vocabulary of technical terms, but we venture to say that very few have ever made a careful study of the organ they undertake to train, such as would be of real service to them in their instructions. A periodical examination of the pupils by a laryngologist would also undoubtedly be of great assistance in order to determine whether the vocal organs were being developed naturally and without any undue strain.

Professor Lewis A. Connell, of Cornell University, has made a special study of auscultation of the chest by the naked ear, and finds that certain sounds can be heard better with the naked ear than through the stethoscope. In the case of cardiac murmurs the majority are best heard through the stethoscope, but the reverse is the case with certain high-pitched blowing diastolic murmurs, which may be clearly distinguished by the naked ear, when quite inaudible through the stethoscope. Quite a large number of pulmonary sounds of a faint high-pitched tone are, according to Professor Connell, heard better with the naked ear, and he instances in particular amphoric breathing and the breath sounds at the beginning of pneumonic consolidation or in pleuritic effusion. The explanation offered to account for these differences is that high-pitched sounds are conducted along closed tubes less perfectly than low-pitched ones, and that the deadening effect is increased if the tubes have elastic walls. It is sufficiently well known that certain sounds can also be better distinguished by means of the old wooden stethoscope than by the binaural. Moreover, when the ear is applied directly to the chest, sound is also conveyed through the bones of the skull. With a flexible stethoscope this bone conduction is lost, and with the high-pitched sounds the intensification produced by the stethoscope is not sufficient to compensate for the loss of bone conduction. To those accustomed to the modern binaural instrument, the old wooden tube is strange and difficult to use with advantage, and the naked ear would probably present similar difficulties at first, but the clinician should be skilled in all means of detecting physical signs. The binaural stethoscope is probably more exclusively used in this country than in any other.

